# The thyroid gland and the process of aging

**DOI:** 10.1186/1756-6614-8-S1-A8

**Published:** 2015-06-22

**Authors:** Adam Gesing

**Affiliations:** 1 Department of Oncological Endocrinology, The Medical University of Lodz, Lodz, Poland

## 

The endocrine organs, including the thyroid gland, undergo important functional changes during aging. It is known that the prevalence of thyroid disorders increases with age. Importantly, subclinical disturbances of thyroid function are more frequent than overt diseases in the elderly. Moreover, the clinical course of thyroid diseases in elderly people differs from that observed in younger subjects; namely, symptoms are more subtle and are often attributed to normal aging, and therefore, require special attention in elderly individuals.

One of the subclinical thyroid function disturbances is subclinical hypothyroidism, which is characterized by normal free thyroxine (FT4) and increased thyrotropin (TSH) levels. The prevalence of subclinical hypothyroidism increases with aging and ranges from 3 to 16 % in individuals aged 60 years and older [1]. In contrast to overt hypothyroidism, the subclinical hypothyroidism in elderly subjects is not associated with impairment of physical and cognitive function, depression, metabolic disturbances or poor quality of life [2,3]. Subclinical hypothyroidism is also not associated with the increased overall mortality risk [2]. Moreover, there is not association between subclinical hypothyroidism and incident coronary heart disease (CHD), heart failure (HF) or cardiovascular (CV) mortality [4]. Similarly, total mortality was not increased in subjects with subclinical hypothyroidism, although the risk of CHD events and of CHD mortality increased with TSH levels 10 mU/L or higher [5].

Importantly, a quite high rate of reversion of subclinical hypothyroidism to euthyroidism in individuals aged at least 65 years with lower baseline TSH levels (4.5-6.9 mU/L) and antithyroid peroxidase antibody (TPOAb) negativity (≤ 37 IU/L) was observed [6]. In turn, higher TSH levels and TPOAb positivity were independently associated with lower chance of reversion to euthyroid status; TSH levels ≥10 mU/L were independently associated with progression to overt hypothyroidism [6].

There are obvious indications for overt hypothyroidism treatment. In turn, indications for treatment of subclinical hypothyroidism are still quite controversial. Nevertheless, the replacement therapy with L-thyroxine is not uniformly recommended in elderly people with subclinical hypothyroidism. For example, L-thyroxine replacement therapy did not improve cognitive function in elderly individuals with subclinical hypothyroidism [7]. Moreover, despite improvement of lipid profile due to treatment of L-thyroxine in subclinical hypothyroidism, there is no clear evidence that this beneficial effect can be associated with decreased cardiovascular or all-cause mortality in elderly patients [8].

Intriguingly, thyroid hypofunction, as well as elevated thyrotropin (TSH) levels may contribute to the extended lifespan. A potential contribution of TSH and thyroid hormones to lifespan regulation was observed in the studies performed on thyroid disease-free population of Ashkenazi Jews, characterized by exceptional longevity (centenarians). For example, the higher serum TSH level in these individuals in comparison with the control groups was observed [9]. Thus, increased serum TSH level seems to be associated with extreme longevity [9]. Moreover, two single nucleotide polymorphisms (SNPs) in TSH receptor (TSHR) gene (namely rs10149689 and rs12050077) were associated with increased TSH level in Ashkenazi Jewish centenarians and their offspring [10]. Also, an inverse correlation between FT4 and TSH levels in centenarians was reported [9] which may suggest a potential role of decreased thyroid function in lifespan regulation, leading to extended longevity. The findings obtained in the Leiden Longevity Study actually show the associations between low thyroid activity and exceptional familial longevity [11].

Also in animals, a reduced thyroid function with low levels of T4 seems to be associated with extended longevity [12-14]. A very severe thyroid hypofunction was observed in Ames dwarf (df/df) mice. These animals are characterized by mutations at the Prop-1 (Prophet of pituitary factor 1) gene and demonstrate a lack of growth hormone (GH), prolactin and TSH. These features may unexpectedly contribute to remarkable longevity in Ames dwarf mice [12]. Furthermore, severe hypothyroid Ames dwarfs and mice with targeted disruption of the growth hormone (GH) receptor/GH binding protein gene (GH receptor knockout; GHRKO) with mild thyroid hypofunction, have decreased thyroid follicle size which may explain decreased thyroid hormone levels in these long-lived mutants [15].

In conclusion, the altered thyroid function may play, via different mechanisms [16], a relevant role in lifespan regulation. Namely, decreased thyroid function may lead to extended longevity.
